# Broad-range amplification and sequencing of the *rpoB* gene: a novel assay for bacterial identification in clinical microbiology

**DOI:** 10.1128/jcm.00266-24

**Published:** 2024-06-17

**Authors:** Joanna Małgorzata Bivand, Ruben Dyrhovden, Audun Sivertsen, Marit Gjerde Tellevik, Robin Patel, Øyvind Kommedal

**Affiliations:** 1 Department of Microbiology, Haukeland University Hospital, Bergen, Norway; 2 Department of Clinical Science, University of Bergen, Bergen, Norway; 3 Division of Clinical Microbiology, Department of Laboratory Medicine and Pathology, Mayo Clinic, Rochester, Minnesota, USA; 4 Division of Public Health, Infectious Diseases, and Occupational Medicine, Department of Medicine, Mayo Clinic, Rochester, Minnesota, USA; Universität Münster, Münster, Germany

**Keywords:** *rpoB*, 16S rRNA, Sanger sequencing, broad-range amplification, bacterial identification

## Abstract

The *rpoB* gene has been proposed as a promising phylogenetic marker for bacterial identification, providing theoretically improved species-level resolution compared to the 16S rRNA gene for a range of clinically important taxa. However, its utility in diagnostic microbiology has been limited by the lack of broad-range primers allowing for its amplification from most species with a single PCR assay. Here, we present an assay for broad-range partial amplification and Sanger sequencing of the *rpoB* gene. To reduce cross-reactivity and allow for *rpoB* amplification directly from patient samples, primers were based on the dual priming oligonucleotide principle. The resulting amplicon is ~550 base pairs in length and appropriate for species-level identification. Systematic *in silico* evaluation of a wide selection of taxa demonstrated improved resolution within multiple important genera, including *Enterococcus, Fusobacterium*, *Mycobacterium*, *Streptococcus*, and *Staphylococcus* species and several genera within the *Enterobacteriaceae* family. Broad-range *rpoB* amplification and Sanger sequencing of 115 bacterial isolates provided unambiguous species-level identification for 97 (84%) isolates, as compared to 57 (50%) using a clinical 16S rRNA gene assay. Several unresolved taxonomic matters disguised by the low resolution of the 16S rRNA gene were revealed using the *rpoB* gene. Using a collection of 33 clinical specimens harboring bacteria and assumed to contain high concentrations of human DNA, the *rpoB* assay identified the pathogen in 29 specimens (88%). Broad-range *rpoB* amplification and sequencing provides a promising tool for bacterial identification, improving discrimination between closely related species and making it amenable for use in culture-based and culture-independent diagnostic approaches.

## INTRODUCTION

The use of gene sequencing for bacterial phylogenetic analysis and identification was first proposed in 1965 by Zuckerkandl and Pauling (1). In 1977, Woese and Fox (2) introduced ribosomal RNA (rRNA) genes for these purposes, and the 16S rRNA gene gradually developed into a cornerstone for both phylogenetic studies and bacterial identification. Today, matrix-assisted laser desorption/ionization time-of-flight (Maldi-TOF) mass spectrometry provides rapid, accurate, and low-cost identification of most cultured isolates in clinical laboratories. However, 16S rRNA sequencing remains an important method for the identification of isolates when Maldi-TOF mass spectrometry fails. Moreover, amplification and sequencing of the 16S rRNA gene directly from patient samples (direct 16S rRNA sequencing) remains the most widespread method for broad-range culture-independent bacterial diagnosis. For the description of novel species, inclusion of a GenBank accession number specifically for the 16S rRNA gene is mandatory, along with an accession number for the whole genome sequence. The 16S rRNA gene has well-known disadvantages for bacterial identification. First, most bacteria have multiple copies of the gene, which can differ in sequence (3, 4). When Sanger sequencing is used, this can cause sequence heterogeneity (3, 4), sometimes rendering chromatograms uninterpretable. Second, for some closely related species, the mutation rate is too low to permit their differentiation (5). Inadequate species resolution in a range of clinically important genera is a major limitation. The increasingly complex and ever-evolving bacterial taxonomy, often resulting from splitting former valid taxons into two or more closely related new species, serves to amplify this challenge.

The *rpoB* gene was suggested by Rowland et al. (6) for molecular identification of bacteria as early as 1993, and later studies confirmed its potential as a phylogenetic marker at the species and genus levels (3, 4, 7–10). The *rpoB* gene encodes the β subunit of a DNA-dependent RNA polymerase and is universally present in the bacterial kingdom (11). It is a single-copy gene, preventing sequence heterogeneity, and due to a higher mutation rate, generally provides better discrimination between closely related species than the 16S rRNA gene (3, 7, 12). Unfortunately, a lack of highly conserved regions suitable for the design of broad-range primers has limited its practical utility. A few attempts at developing broad-range *rpoB* primers have been reported (7, 13), but they are not truly broad-range and therefore not relevant for clinical microbiology. *rpoB* primers targeting selected genera or groups of genera (e.g., fusobacteria, enterococci, streptococci, and staphylococci, and genera within the *Enterobacteriaceae* family) have been published and used to obtain improved species-level resolution within these genera or groups (14–16).

Due to the lack of highly conserved areas in the *rpoB* gene, broad-range primers need to contain multiple degenerate bases to cover all relevant species. In amplifying the bacterial *rpoB* gene directly from patient samples, such degenerate bases increase cross-reactivity with human DNA, thereby reducing the chance of successful bacterial detection. To alleviate this problem, we designed primers based on the dual priming oligonucleotide (DPO) principle, previously reported to mitigate cross-reactivity in broad-range 16S rRNA primers (17).

The aim of this study was to investigate the use of broad-range partial amplification of the *rpoB* gene as an alternative to broad-range partial amplification of the 16S rRNA gene. The investigation included (i) design of a broad-range *rpoB* PCR assay, (ii) extensive *in silico* evaluation of the taxonomic potential of the resulting amplified *rpoB* DNA, (iii) *rpoB* amplification and Sanger sequencing from a broad selection of bacterial isolates from the diagnostic routine, and (iv) amplification of *rpoB* by PCR followed by Sanger sequencing directly from a selection of clinical specimens (i.e., direct *rpoB* sequencing). All *rpoB* results were compared to the results obtained with a 16S rRNA assay used in routine clinical practice and, when appropriate, bacterial whole genome sequencing.

## MATERIALS AND METHODS

### Design and evaluation of the *rpoB* primers

Reference *rpoB* sequences from a manually curated set of clinically relevant bacteria were aligned in Geneious bioinformatics software version 9.1.7 (Dotmatics, Auckland, New Zealand). Thereafter, several degenerated broad-range *rpoB* gene primers were manually designed and evaluated, before choosing two variant forward primers and one reverse primer (Table 1). The primer design was based on the DPO principle with some modifications (18). Binding sites of the selected primers correspond to *Escherichia coli* ATCC 11775 type strain *rpoB* gene nucleotide positions 1,534 and 2,068, producing 535 bp of amplified DNA, with the size varying from ~529 to ~610 base pairs depending on species. The universality of the *rpoB* primers was checked *in silico* against a wide range of bacterial reference genomes from the NCBI database, representing 124 genera covering a broad taxonomic diversity of clinically important microorganisms (Table S1). Critical mismatches in primer 3′-end segments expected to impair amplification and other significant mismatches are displayed in Table 1. Only a few non-critical single mismatches in the DPO 5′-end segment were observed.

**TABLE 1:** Description of the broad-range *rpoB* primers and listing of species/genera with significant mismatches in the primer-binding area*^a^*

Primer names and species/genera with significant mismatches	Sequence
Forward primer 1^*b*^: RpoB_DPO1-F *Desulfovibrio* spp. *Bilophila wadsworthia* *Corynebacterium* spp. *Corynebacterium otitidis* *Metamycoplasma*^*c*^ spp. *Mycoplasmoides*^*c*^ spp.	5′−TCNCARTTYATGGAYCA−I−I−H−I−AAYCC−3′ 5′−−−−−−−−−−−−−−−−−−−−−−−−−−−−−−T−−3′ 5′−−−−−−−−−−−−−−−−−−−−−−−−−−−−−−T−−3′ 5′−−−−−−−−−−−−−−−−−−−−−−−−−−−−−−T−−3′ 5′−−−−−−−−−−−−−−−−−−−−−−−−−−−−−−G−−3′ 5′−−−−−−−−−−−−−−−−−−−−−−−G−−−−−−−−−3′ 5′−A−−−−−−−−T−A−−−−−−−−−−−−−−−−−−−−3′
Forward primer 2^*b*^: RpoB_DPO2-F	5′−AGNCARTTYATGGAYCA−I−I−H−I−AAYCC−3′
Reverse primer^*b*^: RpoB_DPO-R *Deinococcus* spp. *Lactobacillus gasseri/johnsonii/paragasseri* *Saccharibacteria* (TM7) spp.	5′−CNGCYTGDCKYTKCATRTT−I−I−I−I−CCCAT−3′ 3′−−−−−−−−CGA−−−−−−−−−−−−−−−−−−−−−−−−5′ 3′−−−−−−−−−−−−−−GTG−−−−−−−−−−−−−−−−−−5′ 3′−−−−−−−−−−−−−−−−−−−−−−−−−−−−−−−AG−−5′

^*a*^ In addition, single mismatches at the primer 5′ segments were observed for a few species, but these are unlikely to affect amplification.

^*b*^ The PCR contains one reverse primer and two separate forward primers. For species covered by forward primer RpoB_DPO2-F, no significant mismatches were found.

^*c*^ Previously *Mycoplasma* spp. In the primer sequences, D is A, G, or T; K is G or T; Y is C or T; H is A, C, or T; R is A or G; N is any base; I is deoxyinosine; Underlined letters indicate a locked nucleic acid.

### *In silico* analysis of amplified *rpoB* DNA

Guided by the Clinical and Laboratory Standards Institute (CLSI) guideline, “Interpretive Criteria for Identification of Bacteria and Fungi by Targeted DNA Sequencing” (19), 39 clinically relevant bacterial genera were examined using type strain references and other high-quality references (whole genome references and references included in published taxonomic studies) from the NCBI Nucleotide and WGS databases. Alignments for assessing intraspecies variabilities and genetic distance to the next alternative species were conducted in GenBank using BLAST and the Nucleotide and WGS databases. When necessary, reference clustering was performed using the “Distance tree of results” function.

### Biological material and controls

#### Bacterial isolates

To demonstrate *in vitro rpoB* amplification, 115 bacterial isolates representing 95 different species from 36 genera were studied, all analyzed by Maldi-TOF mass spectrometry Microflex (Bruker Biotyper, Bremen, Germany) using the MBT Compass Library Revision K database. Eighteen isolates were not successfully identified by Maldi-TOF mass spectrometry and were therefore submitted to 16S rRNA gene sequencing as a part of the diagnostic procedure. The remaining 97 represented a range of clinically relevant species, including some known to be challenging to identify using 16S rRNA gene sequencing.

#### Clinical samples

To evaluate the amplification of *rpoB* from a background of human DNA, remnant extracted DNA from 33 clinical samples, assumed to contain high levels of human DNA based on sample types, that had previously been investigated with direct 16S rRNA Sanger sequencing were studied. These included 20 heart tissue samples from patients with infective endocarditis, 6 abscess samples, 5 synovial fluid samples, and 2 samples from other tissues (bone marrow and tissue from the knee). The samples were representative of sample types frequently considered for broad-range amplification and Sanger sequencing in our lab. The eluate had been stored at −80°C in the Department of Microbiology, Haukeland University Hospital in Bergen, Norway.

#### Positive and negative controls

Positive and negative extraction controls were included with all PCR setups (Table S2). The positive control was a suspension of *Enterococcus faecalis* ATCC 51559 in sterile water. The negative control for bacterial isolates consisted of the same sterile water used to suspend the bacterial colonies. The negative controls for clinical specimens consisted of PCR-grade water and the same bacterial lysis buffer used for the pretreatment of the clinical sample material as described previously (17).

### Broad-range amplification and Sanger sequencing of *rpoB*

#### DNA extraction

For Sanger sequencing of bacterial isolates, colonies were harvested from agar plates, suspended in 500 µL of sterile water, and incubated for 15 min at 95°C–99°C. After centrifugation for 5 min at 15,550 rcf, the supernatant was directly used as a PCR template. Extraction of bacterial DNA from clinical samples was performed in routine clinical practice, as described previously (17).

#### PCR conditions

PCR was carried out using a QuantStudio5 Real-Time PCR System (ThermoFisher Scientific). The *rpoB* PCR mixture (25 µL) contained 12.5 µL TB Green Premix ExTaq (Takara Bio, Kusatsu, Japan), 2.5 µL of each forward primer, 3 µL of reverse primer, 2.5 µL PCR-grade water, and 2 µL of DNA. All primers are from 10 µM stock solutions. The three-step PCR program included an initial denaturation step at 95°C for 20 s followed by 40 cycles of denaturation at 95°C for 10 s, annealing at 55°C for 15 s, and elongation at 72°C for 20 s. PCR products were cleaned up using the ExoSAP-IT enzymatic degradation kit (Affymetrix, Santa Clara, CA, USA). Thereafter, the product was diluted (1:10) prior to cycle sequencing. Cycle sequencing was carried out in ProFlex PCR System (Applied Biosystems). Amplification mixtures (10 µL) contained 1 µL of PCR product, 2 µL of sequencing buffer (Applied Biosystems) and 1 µL of BigDye (Applied Biosystems). For the *rpoB* gene, 1 µL of each primer and 4 µL of PCR-grade water were used for the forward reaction. For the reverse reaction, 1 µL of primer and 5 µL of PCR-grade water were used. Cycle sequencing thermal conditions included an initial denaturation step at 96°C for 1 min followed by 28 cycles of denaturation at 96°C for 10 s, primer annealing at 60°C for 5 s, and DNA elongation at 60°C for 4 min. Sanger sequencing was performed using an ABI 3730 instrument.

#### Interpretation of chromatograms

Sanger chromatograms were imported to ChromasPro 2.0.1 software (Technelysium Pty Ltd, South Brisbane, Australia) for visualization, trimming of primer sequences, and merging of forward and reverse sequences. Thereafter, *rpoB* consensus sequences for each isolate were assessed using a standard GenBank BLAST search against the Nucleotide and WGS databases (http://blast.ncbi.nlm.gov).

#### Interpretative criteria for *rpoB* sequence analysis

As general criteria for species delineation based on the *rpoB* gene, ≥98.5% homology with a high-quality reference and a minimum distance of ≥1.4% to the next alternative species were applied. Using the “Distance tree of results” function in the GenBank BLAST result page, a lower homology (≥97%) or a shorter distance to the next alternative identification (≥1%) was considered acceptable if three or more references were available for the best matching species and the query sequence formed a well-defined cluster with these clearly separated from neighboring clusters.

For the *Streptococcus mitis* group, separate criteria were established. For *Streptococcus oralis* and *Streptococcus infantis,* interpretative rules proposed by Dyrhovden et al. (14) (≥97.0% homology with a high-quality reference and minimum distance >2.0% to the next alternative species) were applied. For *Streptococcus mitis* and *Streptococcus pseudopneumoniae,* each forming multiple tight clusters (20), high homology to a cluster (≥99%) was accepted for species-level identification, whereas lower homology (97%–99%) was considered sufficient for only a slashed species-level identification (i.e., *S. mitis/pseudopneumoniae*). For *Streptococcus pneumoniae,* where all references formed a single tight cluster (20), an homology ≥99.8% with a high-quality reference was considered sufficient for valid species-level identification.

### Comparator methods

#### Amplification and Sanger sequencing of the 16S rRNA gene

For amplification and Sanger sequencing of the 16S rRNA gene, the DPO primers described by Kommedal et al. (17) with 5′-end modifications later described by Dyrhovden et al. (15) (16S_DPO_Short-F: AGAGTTTGATCMTGGCTCAIIIIIAACGCT and 16S_DPO_Short-R: CGGCTGCTGGCAIIIAITTRGC) were used. Primer binding sites correspond to nucleotide positions 8 and 528 on the 16S rRNA gene of *Escherichia coli* ATCC 11775 (type strain) giving an amplicon size of 520 bp covering the variable areas V1–V3. PCR was carried out using a QuantStudio5 Real-Time PCR System (ThermoFisher Scientific). The 16S rRNA PCR mixture (25 µL) contained 12.5 µL TB Green Premix ExTaq (Takara), 1 µL of each primer (from 10 µM solutions), 8.5 µL nuclease-free water, and 2 µL DNA. The three-step PCR program included an initial denaturation step at 95°C for 20 s followed by 40 cycles with denaturation at 95°C for 10 s, annealing at 60°C for 15 s, and elongation at 72°C for 20 s. Thereafter, cycle sequencing and Sanger sequencing were performed as described for the *rpoB* gene. Interpretation of results generally followed recommendations provided by the CLSI (19), except that, for novel species and for genera or groups of species that have undergone significant taxonomic changes since the publication of the CLSI guidelines, evaluation was performed by the authors per their clinical protocol.

#### Whole-genome sequencing

Whole-genome sequencing was used as a gold standard for the taxonomic assignment of bacterial isolates with discrepant or uncertain results from the 16S rRNA and *rpoB* gene sequencing. Briefly, library preparation was performed according to the Nextera XT DNA Library Preparation Kit (Illumina). Obtained libraries were sequenced on an Illumina MiSeq System (Illumina) using 150 bp paired-end reads. Average nucleotide identity (ANI) was calculated using the ANI Calculator (https://www.ezbiocloud.net/tol/ani). According to accepted criteria (21), ANI ≥95% was used as a cut-off for species delineation, with some exceptions. For the *Streptococcus mitis* group, taxonomy and ANI boundaries proposed by Jensen et al. (22) were used.

## RESULTS

### *In silico* analysis of amplified *rpoB* gene

The *rpoB* gene generally provided improved resolution within genera, whereas the 16S rRNA gene displayed limited resolution to the species level. Results of *in silico* analysis of the *rpoB* amplicon are shown in Table 2. For 28 (71.8%) of the 39 examined genera, the partial *rpoB* gene sequence provided a good resolution to the species level, and for the other 11 (28.2%) genera, some resolution to species level. Moreover, the *rpoB* gene made it easier to identify both acknowledged and unacknowledged taxonomic issues. For example, the *rpoB* gene differentiated well between *Bacteroides fragilis* division I and II, which fulfill current criteria for being reclassified as separate species (23) and showed that *Prevotella melaninogenica* forms two distinct clusters that, based on associated whole genome references, likely represent separate species (ANI 87.9%). Another species with two well-separated *rpoB* variants was *Bacteroides pyogenes*. One population represented references for the abandoned species *Bacteroides tectus*, which was merged into the species *B. pyogenes* in 2010 (24). A 16S rRNA gene homology of only 98.7% combined with an ANI of 94.8% indicates that these might indeed be two separate species and that the former taxonomic classification may have been correct, in concurrence with suggestions from others (25, 26).

**TABLE 2:** Appropriateness of the *rpoB* and 16S rRNA gene for identification of clinically important taxa

Microorganism or group	Appropriateness of the *rpoB* gene _~_550 bp^*a*^	Comments about the *rpoB* gene	Appropriateness of the 16S rRNA gene V1–V3 region^*b*^	Comments about the 16S rRNA gene^*c*^
*Acinetobacter*	Resolution to genus and species.		Resolution to genus, with some resolution to species.	
*Actinomyces*	Resolution to genus and species^*d*^.		Resolution to genus and some species.	Difficult to differentiate between *Actinomyces naeslundii/ Actinomyces viscosus/Actinomyces oris*. Good resolution for other *Actinomyces*.
*Aggregatibacter*	Resolution to genus and usually to species.	Some *Aggregatibacter aphrophilus* strains cannot be differentiated from *Aggregatibacter kilianii*.	Resolution to genus and species.	*A. aphrophilus/A. kilianii/Aggregatibacter segnis/Aggregatibacter actinomycetemcomitans* can be differentiated.
*Bacillus*	Resolution to genus, with some resolution to species.	*Bacillus anthracis/Bacillus tropicus/Bacillus pacificus/Bacillus fungorum/Bacillus paranthracis* cannot be differentiated. *Bacillus cereus/Bacillus thuringiensis* cannot be differentiated. Also, poor resolution between some other species.	Resolution to genus but not to species.	
*Bacteroides*	Resolution to genus and species.	Excellent resolution between *B. fragilis* divisions I and II^*b*^.	Resolution to genus and species.	Differentiation between *B. fragilis* divisions I and II may be possible.
*Bilophila*	Resolution to genus and species.		Resolution to genus but not to species.	Resolution to genus and species.
*Burkholderia*	Resolution to genus and usually to species.	*Burkholderia mallei/Burkholderia pseudomallei* cannot be differentiated.	Resolution to genus, with limited resolution to species.	
*Campylobacter*	Resolution to genus and some to species.	*Campylobacter jejuni/Campylobacter coli* cannot be differentiated.	Resolution to genus and some to species.	*C. jejuni/C. coli/Campylobacter lari/Campylobacter subantarcticus* cannot be differentiated.
*Citrobacter*	Resolution to genus and some to species.	*Citrobacter freundii/Citrobacter youngae/Citrobacter portucalensis/Citrobacter braakii* cannot be differentiated.	Resolution to genus and species.	*C. freundii/C. youngae/Citrobacter pasteurii/C. portucalensis/C. braakii* cannot be differentiated.
*Clostridium*	Resolution to genus and species.	Also, resolution to Groups I–VI for botulinum neurotoxin producing species. Good resolution within *Clostridium novyi sensu lato* cluster.	Resolution to genus but not to species.	Also, resolution to Groups I–VI for botulinum neurotoxin producing species. Uncertain resolution within *C. novyi sensu lato* cluster.
*Cronobacter*	Resolution to genus and usually to species.	*Cronobacter malonaticus/Cronobacter sakazakii* cannot be differentiated.	Resolution to genus and species.	*C. malonaticus/C. sakazakii* cannot be differentiated. *Cronobacter turicensis/Cronobacter universalis* cannot be differentiated.
*Dialister*	Resolution to genus and species.		Resolution to genus but not for all species.	Resolution to genus and species.
*Eikenella*	Resolution to genus and species.		Resolution to genus and species.	*Eikenella corrodens/Eikenella halliae/Eikenella exigua* cannot be differentiated.
*Enterobacter*	Resolution to genus and usually to species^*d*^.	*Enterobacter bugandensis/Enterobacter quasihormaechei* cannot be differentiated.	Resolution to genus with some resolution to species.	Poor resolution to species.
*Enterococcus*	Resolution to genus and species.		Resolution to genus and usually to species.	*Enterococcus faecium/Enterococcus hirae/Enterococcus durans* cannot be differentiated. *Enterococcus casseliflavus/Enterococcus gallinarum* cannot be differentiated.
*Escherichia/Shigella*	Resolution to genus, with some resolution to species.	*E. coli*/*Shigella* cannot be differentiated. Other *Escherichia* spp. can be differentiated.	*E. coli* and *Shigella sonnei* cannot be differentiated.	*E. coli*/*Escherichia furgusonii/Shigella* spp. cannot be differentiated.
*Finegoldia magna*	Resolution to genus and species.		Resolution to genus.	Resolution to genus and species.
*Fusobacterium* ^*e*^	Resolution to genus and species.		Resolution to genus but not to species.	*Fusobacterium polymorphum/Fusobacterium hwasookii/Fusobacterium canifelium* cannot be differentiated. *Fusobacterium periodonticum/Fusobacterium pseudoperiodonticum* cannot be differentiated. Other *Fusobacterium* spp. can be differentiated.
*Haemophilus*	Resolution to genus and species.		Resolution to genus, with some resolution to species.	Poor differentiation between *Haemophilus seminalis* and *Haemophilus haemolyticus*.
*Kingella*	Resolution to genus and species.		Resolution to genus and species.	
*Klebsiella*	Resolution to genus and species.		Fair resolution to genus, with limited resolution to species.	Often not possible to differentiate between *Klebsiella/Enterobacter/Pantoea/ Kosakonia/Yokenella/Pseudescherichia* and *Leclercia*.
*Moraxella*	Resolution to genus and species.		Resolution to genus and species.	
*Mycobacterium tuberculosis*	Resolution to genus and species.	The *M. tuberculosis* complex is merged into one species.		According to current taxonomy the *M. tuberculosis* complex is merged into a single species that can be identified based on 16S rRNA gene.
*Mycobacterium avium* complex (MAC)	Resolution to genus and species.		Resolution of the MAC group into *M. avium* and related species and *Mycobacterium intracellulare* and related species (*M. intracellulare* group, but not to species).	
*Mycobacterium fortuitum* complex	Resolution to genus, with some resolution to species.	*Mycobacterium fortuitum/Mycobacterium houstonense* cannot be differentiated. *Mycobacterium farcinogenes/Mycobacterium senegalense/Mycobacterium conceptionense* cannot be differentiated. *Mycobacterium porcinum/Mycobacterium boenickei* cannot be differentiated.	Resolution to genus, with some resolution to species.	*M. houstoenense/M. farcinogenes/M. senegalense/M. conceptionense/M. fortuitum* cannot be differentiated. *M. porcinum/Mycobacterium boenickei/Mycobacterium neworleansense* cannot be differentiated. *Mycobacterium peregrinum/Mycobacterium septicum/Mycobacterium lutetiense/Mycobacterium montmartrense* cannot be differentiated.
*Mycobacterium simiae* complex	Resolution to genus and species.		Resolution to genus, with some resolution to species	*Mycobacterium triplex/Mycobacterium montefiorense/Mycobacterium florentinum/Mycobacterium stomatepiae/Mycobacterium sherrisii* cannot be differentiated. *Mycobacterium europaeum/Mycobacterium simiae* cannot be differentiated. *Mycobacterium simiae/Mycobacterium shigaense/Mycobacterium paraense* cannot be differentiated. *Mycobacterium lentiflavum/Mycobacterium palustre* cannot be differentiated.
*Mycobacterium kansasii* and related species	Resolution to genus and species.		Resolution to genus, with poor resolution to species.	*M. kansasii/Mycobacterium gastri/Mycobacterium innocens* cannot be differentiated. *Mycobacterium pseudokansasii/Mycobacterium attenuatum/Mycobacterium persicum* cannot be differentiated.
*Mycobacterium* others*f*	Resolution to genus and usually to species.	*Mycobacterium marinum/Mycobacterium ulcerans* cannot be differentiated. *Mycobacterium phocaicum/Mycobacterium mucogenicum* cannot be differentiated.	Resolution to genus and usually to species.	*Mycobacterium marinum/Mycobacterium ulcerans* cannot be differentiated. *Mycobacterium neoaurum/Mycobacterium bacteremicum* cannot be differentiated. *Mycobacterium szulgai/Mycobacterium angelicum/Mycobacterium bohemicum/Mycobacterium riyadhense/Mycobacterium malmoense* cannot be differentiated. *Mycobacterium scrofulaceum/Mycobacterium paraffinicum/Mycobacterium mantenii* and *Mycobacterium paraseoulense* cannot be differentiated. *Mycobacterium phocaicum* and *Mycobacterium mucogenicum* cannot be differentiated.
*Mycobacterium abscessus*	Resolution to genus and species.	Resolution to subspecies.	Resolution to species, but no resolution to subspecies.	*M. abscessus* subsp. *abscessus/M. abscessus subsp. bolletii/M. abscessus* subsp. *massiliense* cannot be differentiated.
*Mycobacterium chelonae* complex	Resolution to genus and species.		Resolution to complex, but no resolution to species.	*M. chelonae/Mycobacterium franklinii/Mycobacterium saopaulense/Mycobacterium salmoniphilum/Mycobacterium stephanolepidis* cannot be differentiated.
*Neisseria*	Resolution to genus and species.		Resolution to genus and usually to species.	*Neisseria meningitidis/ Neisseria cinerea/Neisseria polysaccharea* cannot be differentiated. *Neisseria perflava/Neisseria flavescens* cannot be differentiated. *Neisseria macacae/Neisseria mucosa/Neisseria sicca* cannot be differentiated.
*Nocardia*	Resolution to genus and species.		Resolution to genus, with limited resolution to species.	
*Parvimonas*	Resolution to genus and species.		Not included in the CLSI document.	Resolution to genus and species.
*Peptoniphilus*	Resolution to genus and species.		Resolution to genus but not to species within some genera.	Resolution to genus and species.
*Peptostreptococcus*	Resolution to genus and species.		Resolution to genus.	Resolution to species may be possible, genus evolving.
*Porphyromonas*	Resolution to genus and species.		Resolution to genus and species.	
*Prevotella*	Resolution to genus and species.		Resolution to genus but not to species.	Resolution to species.
*Proteus*	Resolution to genus and species.		Resolution to genus and species.	*Proteus vulgaris/Proteus hauseri* cannot be differentiated.
*Pseudomonas*	Resolution to genus, with some resolution to species.	May be difficult to differentiate between some of the environmental species. *Pseudomonas aeruginosa* can be differentiated.	Resolution to genus, with limited resolution to species (except for *P. aeruginosa*).	
*Salmonella*	Resolution to genus and species.		Resolution to genus, with limited resolution to species.	
*Schaalia*	Resolution to genus and species.		Not included in the CLSI document.	Resolution to genus and species.
*Serratia*	Resolution to genus, with some resolution to species.	*Serratia marcescens/Serratia ureilytica/Serratia nematodiphila* cannot be differentiated.	Resolution to genus and species.	*S. marcescens/S. ureilytica/S. nematodiphila* cannot be differentiated.
*Staphylococcus aureus* complex	Resolution to genus and species.		Not included in the CLSI document.	Species within *S. aureus* complex cannot be differentiated.
*Staphylococcus* others	Resolution to genus and species.		Resolution to genus, with limited resolution to species / for some cases usually resolution to species.	*Staphylococcus capitis/Staphylococcus caprae/Staphylococcus epidermidis* cannot be differentiated. *Staphylococcus intermedius/Staphylococcus delphini/Staphylococcus pseudointermedius* cannot be differentiated. *Staphylococcus haemolyticus/Staphylococcus hominis/Staphylococcus caledonicus/Staphylococcus devriesei/Staphylococcus taiwanensis/Staphylococcus borealis* cannot be *differentiated. Staphylococcus warneri/Staphylococcus pasteuri* cannot be differentiated.
*Stenotrophomonas maltophilia*	Resolution to genus and species.		Resolution to genus and species.	
*Streptococcus mitis* group	Resolution to genus and species.		Resolution to genus and group, with poor resolution to species.	*S. mitis/S. pneumoniae/S. pseudopneumoniae* cannot be differentiated.
*Streptococcus salivarius* group	Resolution to genus and species.		Resolution to genus and group, with poor resolution to species.	*S. salivarius/S. thermophilus/S. vestibularis* cannot be differentiated.
*Streptococcus equinus* group	Resolution to genus and species.	Due to limited references for *rpoB*, differentiation to subspecies for *Streptococcus gallolyticus* may be difficult.	Resolution to genus and group, with poor resolution to species.	*S. gallolyticus* subsp. *gallolyticus/S. gallolyticus* subsp. *macedonicus /S. gallolyticus* subsp. *pasteurianus/Streptococcus equinus/Streptococcus infantarius* can be differentiated.
*Streptococcus mutans* group	Resolution to genus and species.		Resolution to genus and group, with good resolution of most species.	
*Streptococcus anginosus* group	Resolution to genus and species.		Resolution to genus and species.	
β-hemolytic *Streptococcus*	Resolution to genus and species.		Resolution to genus and species.	
*Veillonella*	Resolution to genus and usually to species.	*Veillonella nakazawae/Veillonella infantium* cannot be differentiated.	Resolution to genus but not for all species.	*Veillonella dispar/Veillonella parvula/Veillonella nakazawae/Veillonella infantium* cannot be differentiated.

^*a*^ Based on the *in silico* analysis and sequence results from this study.

^*b*^ According to the CLSI document.

^*c*^ Based on the CLSI document and findings from this study.

^*d*^ For some species, resolution is based on the cut-off and/or clustering in the NCBI database.

^*e*^ Multiple inaccuracies in the NCBI database for *Fusobacterium nucleatum* group references. Many references representing *Fusobacterium animalis*, *F. polymorphum,* or *Fusobacterium vincentii* have been submitted as *F. nucleatum*.

^*f*^ Other clinically relevant species within *Mycobacterium* genera that are not mentioned here can usually be separated to species level with the *rpoB* gene.

Unusually high variability and multiple distinct *rpoB* clusters were observed within several species without evidence of being hybrid taxons; these included *Aggregatibacter aphrophilus, Streptococcus intermedius,* and species in the *Streptococcus mitis* group. For *A. aphrophilus*, valid *rpoB* gene references formed three distinct clusters [similar to clusters described in a recent WGS taxonomy study (27)], with 92.7%–96.7% intercluster homologies. One of these clusters was similar to the *rpoB* gene of *Aggregatibacter kilianii*, limiting discrimination between *A. kilianii* and *A. aphrophilus* and some strains of *A. aphrophilus* from *A. kilianii*. Also, *S. intermedius* was found to have three distinct *rpoB* gene variants, all well-separated from other species in the *S. anginosus* group.

In the *Streptococcus mitis* group, *Streptococcus oralis* formed multiple distinct *rpoB* clusters with high intra-cluster homology and typically a 2.7% distance to the closest neighboring clusters. All clusters grouped together on a common *S. oralis* branch clearly separated from other species in the *S. mitis* group. The overall *rpoB* intra-species variability for *S. oralis* was found to be ~6%. The *rpoB* references for *S. mitis* and *S. pseudopneumoniae* clustered in the same manner as observed for *S. oralis* with overall intraspecies variabilities of 5% and 4%, respectively. However, for these two species, clusters were intermixed at the same phylogenetic main branch as also observed by Jensen et al*.* (20). We, therefore, suggest that for *S. mitis* and *S. pseudopneumoniae*, the *rpoB* gene can be used for species-level identification if treated like a barcode instead of a phylogenetic chromometer [i.e., very high homology to a cluster (>99%) can be used to infer species-level identification with lower homology (97%–99%) allowing group-level identification only (*S. mitis*/*pseudopneumoniae*) regardless of the closest match]. *Streptococcus pneumoniae,* a highly specialized strict human pathogen, was found to form a single cluster with a 3% distance to the closest neighboring clusters representing *S. mitis* and *S. pseudopneumoniae*. Provided the high number of references available for *S. pneumoniae* combined with the very high homology within the *S. pneumonia*e cluster (>99.4%), we suggest that species-level identification can be assigned for sequences having a homology ≥99.8% with a high-quality *S. pneumoniae* reference.

### Sanger sequencing of bacterial isolates

**Fig 1:**
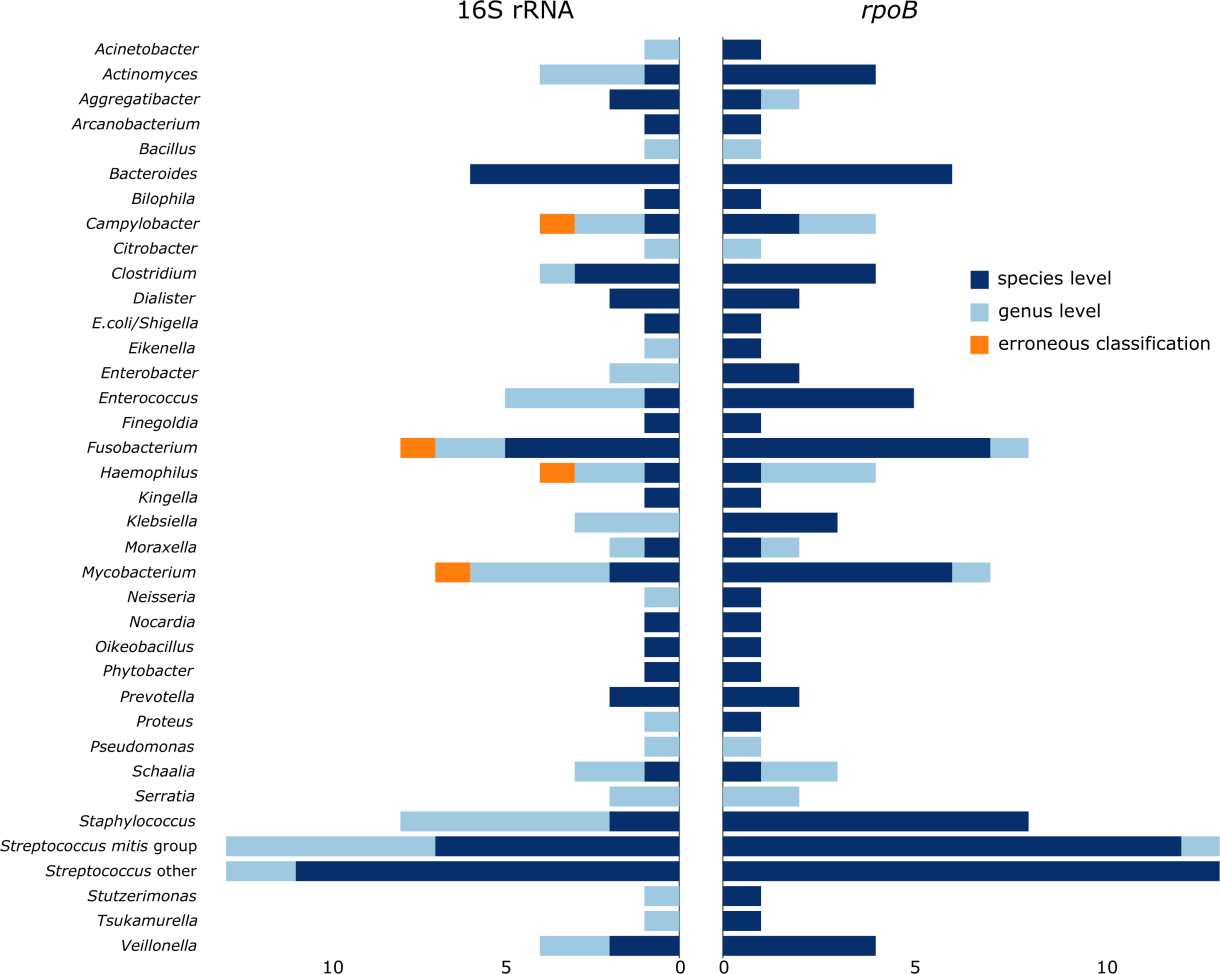
Results from 16S rRNA gene and *rpoB* amplification and Sanger sequencing of 115 bacterial isolates. Colors indicate taxonomic classification level. Numbers below the figure indicate the number of included isolates from each genus/genus group.

*rpoB* sequencing provided species-level identification for 97 (84%) of 115 isolates, as compared to 57 (50%) for partial 16S rRNA gene sequencing (Fig. 1). The main rule for distance to next (≥1.4%) was not met for *Actinomyces naeslundii* versus *Actinomyces oris*, but references from the WGS database formed two distinct clusters (using the “Distance tree of results” function) supporting that differentiation is possible. The same applied for distinction between *Enterobacter ludwigii* and *Enterobacter wuhouensis*. One isolate of *Veillonella montpellierensis* did not meet the criteria for species-level homology (≥98.5%), albeit still clustering with the *V. montpellierensis* references in the WGS database, well separated from other species. It is therefore likely that the low homology score was related to missing variant references in the database and that species identification could be achieved. A similar situation was observed for an isolate of *Aggregatibacter segnis*. Both results were supported by WGS results (Table 3).

**TABLE 3:** Results of whole-genome sequencing of 17 bacterial isolates^*i*^

ID	16S rRNA results	%ID best^*a*^	%ID type^*b*^	*rpoB* results	%ID best^*a*^	%ID type^*b*^	WGS match with type strain material^*c*^	ANI (%)	Conclusion^*d*^
5	***Actinomyces oralis***	**98.4**	**98.4**	*A. oralis*	100	100	*A. oralis*	98.5	*A. oralis*
	*Actinomyces naeslundii*	99.0	98.8	*A. naeslundii*	98.6	98.1	***A. naeslundii***	**88.2**	
7	*Aggregatibacter aphrophilus*	99.8	99.8	*A. aphrophilus*	99.0	93.2	*A. aphrophilus*	97.0	*A. aphrophilus*
				*A. kilianii*	99.6	98.6	***A. kilianii***	**94.3**	
8	*Aggregatibacter segnis*	100	100	***A. segnis***	**97.5**	**96.3**	*A. segnis*	96.2	*A. segnis*
22	*Campylobacter showae*	100	99.6	*C. rectus*	99.4	99.2	*C. rectus*	96.1	*C. rectus*
	*Campylobacter rectus*	93.9	93.9	***C. showae***	**93.5**	**93.5**	***C. showae***	**89.0**	
46	***Fusobacterium animalis***	**98.0**	**97.8**	***F. animalis***	**96.8**	**96.0**	***F. animalis***	**93.0**	*Fusobacterium* sp.^*g*^
48	***Haemophilus haemolyticus***	**98.9**	**98.3**	***H. haemolyticus***	**97.7**	**96.7**	***H. haemolyticus***	**94.9**	*Haemophilus* sp.^*g*^
	***Haemophilus seminalis***	**100**	**99.8**	***H. seminalis***	**96.5**	**96.5**	***H. seminalis***	**92.5**	
50	*Haemophilus parainfluenzae*	97.9	97.2	***H. parainfluenzae***	**97.7**	**95.3**	***H. parainfluenzae***	92.4	*Haemophilus* sp.^*g*^
51	***Haemophilus haemolyticus***	**97.9**	**96.2**	***H. haemolyticus***	**97.9**	**97.7**	***H. haemolyticus***	94.2	*Haemophilus* sp.^*g*^
57	*Moraxella osloensis*	99.7	99.4	**M. osloensis**	**97.7**	96.5	*M. osloensis*	95.0	*Moraxella* sp.^*g*^
	*Moraxella tetraodonis*	99.7	99.7	*M. tetraodonis*	99.4	99.4	*M. tetraodonis*	95.7	
	*Moraxella osloensis* YV1^*e*^	99.7	–^*f*^	*M. osloensis* YV1^*e*^	99.8	–^*f*^	*M. osloensis* YV1^*e*^	94.9	
62	*Mycobacterium bacteremicum*	100	99.8	***M. bacteremicum***	**93.8**	**93.2**	***M. bacteremicum***	**77.0**	*Mycobacterium* sp.^*g*^
	*Mycobacterium neoaurum*	100	100	***M. neoaurum***	**93.0**	**92.4**	***M. neoaurum***	**76.5**	
72	***Schaalia odontotylica***	**98.9**	**98.0**	***S. odontolytica***	**97.1**	**94.1**	***S. odontolytica***	**85.9**	*Schaalia* sp.^*g*^
73	***Schaalia turicensis***	**97.8**	**97.4**	***S. turicensis***	**97.7**	–^*f*^	*S. turicensis* ^*e*^ ^,^ ^*f*^	95.8	*S. turicensis*
88	***Streptococcus infantis***	**98.7**	**98.7**	*S. infantis*	97.7	97.3	*S. infantis*	93.6	*S. infantis* ^*h*^
89	*Streptococcus intermedius*	100	100	*S. intermedius*	99.6	93.4	*S. intermedius*	98.4	*S. intermedius*
95	*Streptococcus oralis*	100	99.6	*S. oralis*	97.7	95.9	*S. oralis*	94.9	*S. oralis* ^*h*^
96	*Streptococcus oralis*	100	99.6	*S. oralis*	97.3	96.1	*S. oralis*	95.3	*S. oralis*
112	*Veillonella montpellierensis*	99.8	99.8	***V. montpellierensis***	**96.5**	**96.5**	*V. montpellierensis* ^*e*^ ^,^ ^*f*^	96.1	*V. montpellierensis*

^*a*^ Results from Sanger sequencing showing best %ID match.

^*b*^ Results from Sanger sequencing showing %ID match with type strain.

^*c*^ Results from WGS and %ANI (best match) with type strain (the same type strain as for Sanger sequencing results’ analysis).

^*d*^ Summarized results showing final identification.

^*e*^ Not type material.

^*f*^ Type material not available.

^*g*^ ANI supports possible new, separate species.

^*h*^ Special criteria used for *Streptococcus mitis* group.

^*i*^ Bold text—identification to species level not met according to accepted criteria.

Based on the *rpoB* amplicon, species-level identification was obtained for six of seven *Mycobacterium* isolates, as compared to two out of seven with 16S rRNA gene sequencing. The single isolate that could not be identified based on *rpoB* sequencing was reported as *Mycobacterium neoaurum/bacteremicum* based on a 100% 16S rRNA gene homology with both type strains. Partial *rpoB* gene homologies with these species were only 92.4% and 93.2%, respectively; WGS confirmed the isolate to represent a novel species (highest ANI 77% with *M. bacteremicum*).

For the 13 isolates from the *S. mitis* group, species-level identification was obtained for 12 by *rpoB* sequencing (Fig. 1). In three of four isolates, separation of *S. mitis*, *S. pneumoniae,* and *S. pseudopneumoniae* was achieved. In one isolate, the percent identity with *S. mitis* (98.4%) was too low for species assignment according to the applied *S. mitis*/*S. pseudopneumoniae* criteria, resulting in identification as *S. mitis/S. pseudopneumoniae*.

One of the *Campylobacter* isolates had a 99.4% *rpoB* identity with *Campylobacter rectus* and 93.5% with *Campylobacter showae*, while the 16S rRNA gene yielded clear-cut identification of *C. showae* with a homology with *C. rectus* of only 93.9%. Repeat analysis confirmed these discrepant results. Maldi-TOF mass spectrometry supported the *rpoB*-based identification, and WGS showed a 96.1% ANI with *C. rectus* and only 89% ANI with *C. showae*.

Neither *rpoB* nor 16S rRNA gene sequencing discriminated between *Moraxella osloensis*, *Moraxella osloensis* strain YV1, and *Moraxella tetraodonis*. Strain YV1 might represent a novel species separate from *M. osloensis* (ANI with type strain, 94.9%). WGS of our *Moraxella* isolate also yielded ambiguous results (Table 3), indicating unresolved taxonomic issues in the *Moraxella* genus and resulting in genus-level identification only.

*rpoB* sequencing differentiated between all known *Fusobacterium* species and identified seven of eight *Fusobacterium* isolates to the species level. The last isolate yielded a valid 16S rRNA gene result of *Fusobacterium animalis* (99.3%) with a *rpoB* identity of only 96.8%. WGS showed that this is probably a new species (ANI 93% with *F. animalis*) and that the species-level identification inferred by the 16S rRNA gene was incorrect. 16S rRNA gene Sanger sequencing provided correct species identification for four *Fusobacterium* isolates.

Amplified DNA sequencing retrieved ambiguous results for 17 isolates. For these isolates, WGS aligned with *rpoB* yielded identification for 16 (Table 3). For the last isolate (ID = 57), WGS-based identification was inconclusive. Five isolates (IDs = 22, 46, 48, 50, and 62) were incorrectly classified based on 16S rRNA gene sequencing. Four were likely novel species sharing >99% homology with other already described species (Table 3).

### Direct *rpoB* sequencing of clinical samples

The *rpoB* gene was successfully amplified and sequenced from 29 (88%) of 33 clinical samples (Table 4). Due to cross-reactivity with human DNA, chromatograms were uninterpretable from four samples (all with *rpoB* PCR Ct-values > 30). For four additional samples, human DNA was present in the chromatograms but did not prevent bacterial identification (Table 4). Sequencing of the 16S rRNA gene gave clean chromatograms in both forward and reverse directions in all 33 cases. The *rpoB* gene provided species-level resolution in 27/29 (93.1%) samples compared to 17/33 (51.5%) with the 16S rRNA gene. For 32 (97%) samples, the Ct-value for the *rpoB* PCR was higher than that of the 16S rRNA PCR (average 5.9 cycles, range 2.0–9.4) (Fig. S1).

**TABLE 4:** Results from *rpoB* and 16S rRNA gene amplification and Sanger sequencing directly from clinical samples

ID	Sample type	16S rRNA	*rpoB*
1	Endocarditis	*Streptococcus mutans*	*Streptococcus mutans*
2	Endocarditis	*Aggregatibacter actinomycetemcomitans*	*Aggregatibacter actinomycetemcomitans*
3	Endocarditis	*Staphylococcus aureus* complex	*Staphylococcus aureus*
4	Endocarditis	*Streptococcus gordonii*	*Streptococcus gordonii*
5	Endocarditis	*Streptococcus gordonii*	*Streptococcus gordonii*
6	Endocarditis	*Enterococcus faecalis*	*Enterococcus faecalis*
7	Endocarditis	*Streptococcus sanguinis*	*Streptococcus sanguinis*
8	Endocarditis	*Streptococcus mitis* group	*Streptococcus mitis/pseudopneumoniae*
9	Endocarditis	*Streptococcus sanguinis*	*Streptococcus sanguinis*
10	Endocarditis	*Haemophilus parainfluenzae*	*Haemophilus parainfluenzae*
11	Endocarditis	*Streptococcus salivarius* group	Human DNA
12	Bone marrow	*Clostridium novyi sensu lato*	*Clostridium novyi* B lineage II*^a^*
13	Endocarditis	*Streptococcus mitis* group	*Streptococcus pneumoniae*
14	Abscess, liver	*Fusobacterium animalis*	*Fusobacterium animalis*
15	Synovial fluid, knee	*Streptococcus* species	*Streptococcus infantis*
16	Synovial fluid, hip prothesis	*Bacteroides fragilis*	*Bacteroides fragilis* division I^*a*^
17	Abscess, spondylodiscitis	*Bacteroides fragilis*	*Bacteroides fragilis* division I
18	Abscess, hip	*Parvimonas micra*	*Parvimonas micra* ^*a*^
19	Abscess, abdominal aorta	*Bacteroides fragilis*	*Bacteroides fragilis* division I
20	Endocarditis	*Streptococcus gallolyticus*	*Streptococcus gallolyticus*
21	Endocarditis	*Tropheryma whipplei*	*Tropheryma whipplei*
22	Synovial fluid, ankle	*Neisseria meningitidis/cinerea/polysaccharea*	*Neisseria meningitidis* ^*b*^
23	Endocarditis	*Enterobacter hormaechei/quasihormaechei*	*Enterobacter hormaechei*
24	Endocarditis	*Streptococcus oralis*	*Streptococcus oralis*
25	Endocarditis	*Streptococcus salivarius* group	*Streptococcus salivarius*
26	Endocarditis	*Streptococcus mitis* group	*Streptococcus mitis*
27	Synovial fluid, shoulder	*Fusobacterium vincentii*	*Fusobacterium vincentii*
28	Endocarditis	*Staphylococcus epidermidis/caprae/capitis*	*Staphylococcus epidermidis*
29	Synovial fluid, hip joint	*Prevotella* species	Human DNA
30	Tissue, knee prothesis	*Enterobacter hormaechei/quasihormaechei/asburiae*	Human DNA
31	Endocarditis	*Staphylococcus caprae/capitis/epidermidis/saccharolyticus*	*Staphylococcus caprae*
32	Abscess, hip prothesis	*Streptococcus mitis* group	*Streptococcus mitis/pseudopneumoniae*
33	Abscess, hip	*Klebsiella pneumoniae/variicola quasipneumoniae/africana*	Human DNA

^*a*^ Only forward or reverse sequence was interpretable due to co-sequencing of human DNA.

^*b*^ Human DNA co-sequenced and visible as lower secondary peaks that did not interfere with interpretation.

## DISCUSSION

In this study, we present the first truly broad-range PCR for partial amplification of the bacterial *rpoB* gene. The amplified fragment of ~550 bp allows microbial identification and provides improved species-level resolution compared to partial amplification of the 16S rRNA gene. DPO-inspired primer design limits cross-reactivity with human DNA and allows for amplification of *rpoB* directly from clinical samples.

As shown by both the *in silico* evaluation and analysis of clinical bacterial isolates, *rpoB* is less tolerant of unresolved taxonomic matters disguised by the lower resolution of the 16S rRNA gene. Among clinical isolates from our diagnostic routine, four putative novel species were erroneously assigned to a known species by 16S rRNA analysis, whereas *rpoB* correctly assigned these to a genus-level only identification (Fig. 1). The use of *rpoB* sequencing in diagnostic microbiology therefore holds the potential to accelerate discoveries of novel clinically relevant species, and inclusion of a separate GenBank reference for the *rpoB* gene is encouraged for description and publishing of new taxa. Correct taxonomic assignments can be clinically important for detections directly from clinical specimens that are ultimately culture negative and therefore cannot be associated with phenotypic susceptibility testing. For instance, the yet not formally named species currently referred to as “*Bacteroides fragilis* genomospecies division II,” harbors the *cfiA* gene encoding meropenem resistance that has not been found in “division I” isolates. While the 16S rRNA gene does not reliably discriminate between the two divisions, the *rpoB* gene clearly does (*rpoB* gene homology ~91%) (23, 28).

The *rpoB* gene contains only a few very short conserved and semi-conserved areas. For broad-range amplification, highly degenerate primers have been used, resulting in unacceptable levels of cross-reactivity with human DNA. To overcome this problem, the DPO principle was used for primer design. The DPO principle offers an additional advantage since it involves a poly-inosine linker that can be used to bridge two adjacent conserved regions separated by a short variable section. Although the *rpoB* gene did not permit strict use of DPO design recommendations, the primers nevertheless significantly reduced cross-reactivity and the PCR performed well on sample types with high concentrations of human DNA. Not all polymerases will tolerate the poly-inosine linker (18). This must be considered when choosing a different master mix from the one used here.

When comparing the novel *rpoB* PCR assay to a 16S rRNA PCR assay, lower Ct-values were found for the latter, as a result of the 16S rRNA gene being present in multiple copies in most species. The multiple 16S rRNA gene copies should in theory reduce Ct-values between one and three cycles depending on the species. However, Ct-values for the *rpoB* PCR were on average almost six cycles higher, suggesting that the *rpoB* PCR used is less efficient than the 16S rRNA gene PCR used. Presumably, reduced efficiency is a consequence of the many variable primer positions, leading to less efficient primer binding.

The number of available *rpoB* gene references is inadequate for many bacteria, in particular for novel species. For some, references are only available in the WGS database. This can be a limitation since this database does not allow for hypothesis-free analysis but requires a definition of the species or genus to search against. However, the results below cut-off from a standard GenBank search or from Maldi-TOF mass spectrometry might guide in the right direction. As demonstrated in this study for *A. aphrophilus* and *V. montpellierensis,* a limited number of references in combination with relatively high intra-species variability may be a reason for insufficient homology scores. The problem with limited databases will dwindle with the rapidly increasing number of available complete genome references.

An inherent limitation of identification based on a single gene is homologous recombination events, where the entire gene or a gene fragment is exchanged with a similar or identical nucleotide sequence from a related species (29). Homologous recombination of the entire *rpoB* gene has been described in several strains of *Mycobacterium intracellulare* subspecies *chimaera* (previously *Mycobacterium yongonense*), which have acquired the *rpoB* gene of *Mycobacterium parascrofulaceum* (30). Homologous recombination of the complete *rpoB* gene was also reported in the study by Jensen et al. (20) for an isolate of *S. pseudopneumoniae* (strain 5247, accession AYRQ01000028.1) that had acquired the *rpoB* gene of *S. pneumoniae*. Among 111 available whole genome references of *S. pseudopneumoniae*, there is only one more isolate (strain BVY9BV, accession JAVPVR010000089.1) where this appears to have occurred. In the present study, based on available whole genome references in the Nucleotide and WGS databases, there was evidence for both complete and partial homologous recombination in the *rpoB* gene of several species. Complete homologous recombination was recognized between *Streptococcus dysgalactiae* subsp. *equisimilis* and *Streptococcus pyogenes* in three references (LS483521.1, CP060639.1, and LR594045.1). These two species are known to undergo genetic exchange in both core and accessory genes (31). Evidence of homologous recombination of large segments of *rpoB* was also found in subpopulations of *S. intermedius* and *A. aphrophilus* (Fig. S2 and S3), but none of these partial recombinations prevented correct identification based on the amplified DNA analyzed in this study. In the context of bacterial detection and identification, the multiple copies of the 16S rRNA gene are generally considered as limitation. However, at least for Sanger sequencing, they can protect against erroneous identifications due to homologous recombination since the dominant peaks in the chromatogram represent the majority of the copies. Interestingly, in this study, a clinical isolate of *C. rectus* erroneously identified as *C. showae* by 16S rRNA gene sequencing was found, despite this species being reported to have three copies of the gene. A full investigation of this isolate will be published in a separate manuscript.

Due to the limitations discussed above, broad-range amplification of *rpoB* cannot replace broad-range amplification of the 16S rRNA gene but should be used as an alternative or a supplement to enhance identifications when appropriate. In broad-range amplification and Sanger sequencing directly from patient samples, all samples should be screened using a more sensitive 16S rRNA PCR, and only moderate to strong positive samples will be candidates for *rpoB* gene analysis. In targeted next-generation sequencing, *rpoB* could be used as a supplement target to the 16S rRNA gene in all samples. In such a dual-target approach, the results from the well-understood 16S rRNA gene can form the backbone for bacterial identifications, supplemented by the higher resolution of the *rpoB* gene (12, 14, 15).

### Conclusion

Broad-range amplification and sequencing of the *rpoB* gene is a promising tool for the identification of bacterial isolates, providing improved differentiation of a range of clinically important species. It can also be used for culture-independent bacterial identification directly from clinical samples (e.g., in patients with endocarditis, an infection sometimes caused by bacteria that cannot be identified to the species level using the 16S rRNA gene). The possibility of homologous recombination and limited references for some species favor using it in combination with the 16S rRNA gene. Such a combination approach is a theoretically attractive approach for targeted metagenomic sequencing.

## Data Availability

All bacterial whole genome sequences were deposited in the European Nucleotide Archive (ENA) at the European Molecular Biology Laboratory-European Bioinformatics Institute (EMBL-EBI) under accession number PRJEB75571 . The 16S rRNA gene and *rpoB* sequences generated in this study are all provided in Table S3.

## SUPPLEMENTAL MATERIAL

Fig. S1 jcm.00266-24-s0001.pdf Ct values from the 16S rRNA gene and *rpoB* PCRs.

Fig. S2 jcm.00266-24-s0002.pdf Homologous recombination of large segment in the *rpoB* gene in subpopulations of *Streptococcus intermedius*.

Fig. S3 jcm.00266-24-s0003.pdf Homologous recombination of large segment in the *rpoB* gene in subpopulations of *Aggregatibacter aphrophilus*.

Table S1 jcm.00266-24-s0004.docx Overview of all genera checked *in silico*.

Table S2 jcm.00266-24-s0005.docx Positive and negative controls.

Table S3 jcm.00266-24-s0006.xlsx Sequencing data.

ASM does not own the copyrights to Supplemental Material that may be linked to, or accessed through, an article. The authors have granted ASM a non-exclusive, world-wide license to publish the Supplemental Material files. Please contact the corresponding author directly for reuse.
