# Supplementary material for: Broad-range amplification and sequencing of the rpoB gene: a novel assay for bacterial identification in clinical microbiology
Source: J Clin Microbiol. 2024 Jun 17;62(7):e00266-24. doi: 10.1128/jcm.00266-24 (PMC11324016; doi:10.1128/jcm.00266-24)
Supplement: Fig. S1 — Ct values from the 16S rRNA gene and rpoB PCRs. [file jcm.00266-24-s0001.pdf]

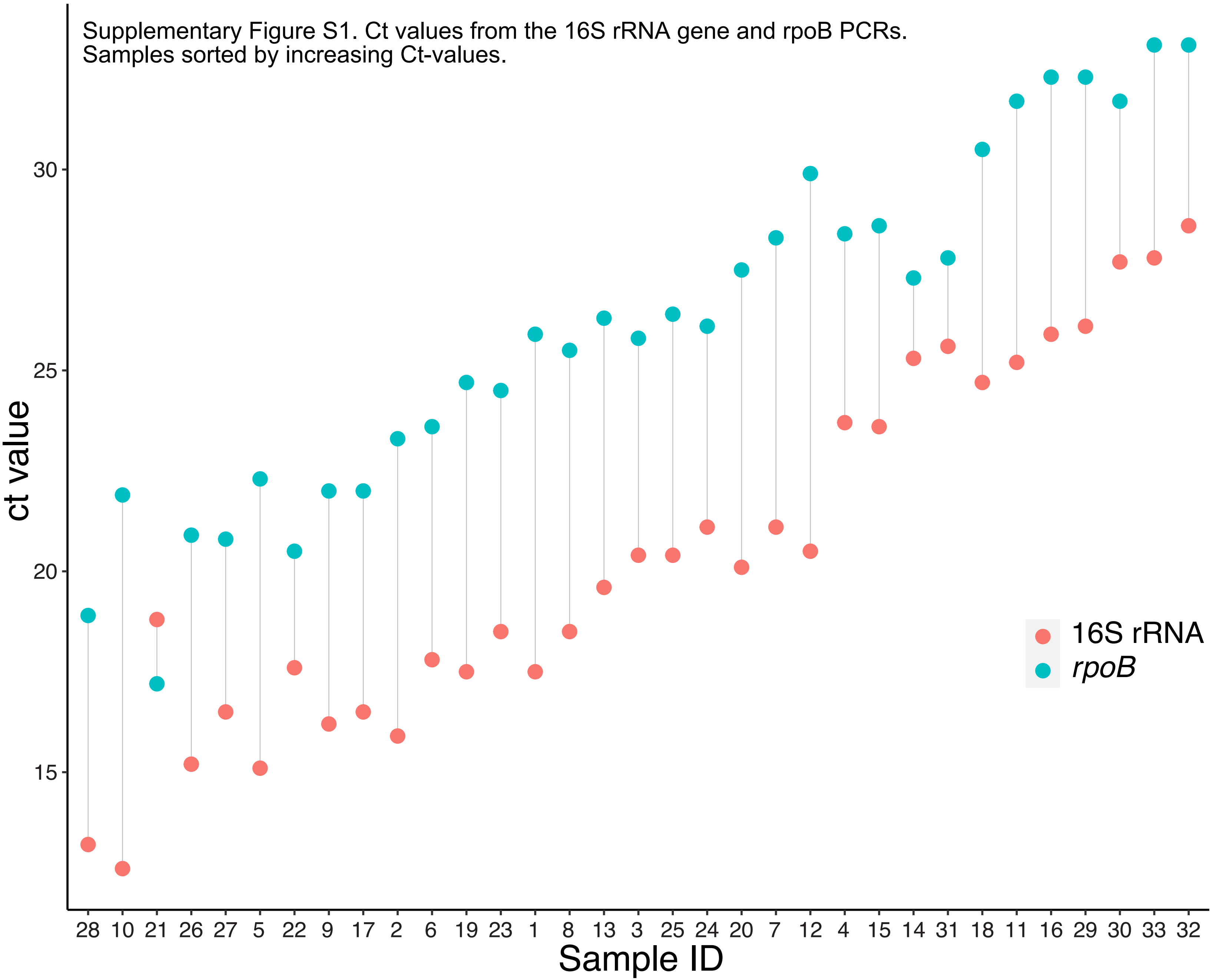

Supplementary Figure S1. Ct values from the 16S rRNA gene and rpoB PCRs.
Samples sorted by increasing Ct-values.
