# Supplementary material for: Broad-range amplification and sequencing of the rpoB gene: a novel assay for bacterial identification in clinical microbiology
Source: J Clin Microbiol. 2024 Jun 17;62(7):e00266-24. doi: 10.1128/jcm.00266-24 (PMC11324016; doi:10.1128/jcm.00266-24)

Supplementary Figure S3. Homologous recombination of large segment in the rpoB gene in subpopulations of Aggregatibacter aphrophilus. Yellow arrows indicate forward and reverse primers positions. Black lines indicate differences in nucleotide sequences.

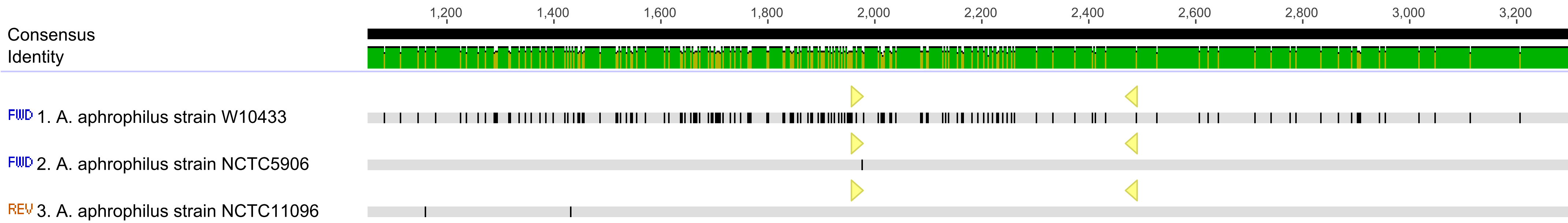

Supplement: Fig. S3 — Homologous recombination of large segment in the rpoB gene in subpopulations of Aggregatibacter aphrophilus. [file jcm.00266-24-s0003.pdf]
