# Supplementary material for: Broad-range amplification and sequencing of the rpoB gene: a novel assay for bacterial identification in clinical microbiology
Source: J Clin Microbiol. 2024 Jun 17;62(7):e00266-24. doi: 10.1128/jcm.00266-24 (PMC11324016; doi:10.1128/jcm.00266-24)
Supplement: Table S1 — Overview of all genera checked in silico. [file jcm.00266-24-s0004.docx]

**SUPPLEMENTARY TABLE S1** Universality of the *rpoB* primers. Overview of all genera checked *in silico*.

| **Genera** | **Forward primer** | **Reverse primer** |
| --- | --- | --- |
| *Abiotrophia* | RpoB_DPO1-F | RpoB_DPO-R |
| *Acinetobacter* | RpoB_DPO1-F | RpoB_DPO-R |
| *Actinobacillus* | RpoB_DPO1-F | RpoB_DPO-R |
| *Actinomyces* | RpoB_DPO1-F | RpoB_DPO-R |
| *Actinotignum* | RpoB_DPO1-F | RpoB_DPO-R |
| *Aerococcus* | RpoB_DPO1-F | RpoB_DPO-R |
| *Aggregatibacter* | RpoB_DPO1-F | RpoB_DPO-R |
| *Agrobacterium* | RpoB_DPO1-F | RpoB_DPO-R |
| *Akkermansia* | RpoB_DPO1-F | RpoB_DPO-R |
| *Alcaligenes* | RpoB_DPO1-F | RpoB_DPO-R |
| *Alistipes* | RpoB_DPO1-F | RpoB_DPO-R |
| *Alloprevotella* | RpoB_DPO1-F | RpoB_DPO-R |
| *Alloscardovia* | RpoB_DPO1-F | RpoB_DPO-R |
| *Anaerococcus* | RpoB_DPO1-F | RpoB_DPO-R |
| *Anaeroglobus* | RpoB_DPO1-F | RpoB_DPO-R |
| *Atopobium* | RpoB_DPO1-F | RpoB_DPO-R |
| *Bacillus* | RpoB_DPO1-F | RpoB_DPO-R |
| *Bacteroides* | RpoB_DPO1-F | RpoB_DPO-R |
| *Bartonella* | RpoB_DPO1-F | RpoB_DPO-R |
| *Bergeriella* | RpoB_DPO2-F | RpoB_DPO-R |
| *Bifidobacterium* | RpoB_DPO1-F | RpoB_DPO-R |
| *Bilophila* | Mismatch | RpoB_DPO-R |
| *Blautia* | RpoB_DPO1-F | RpoB_DPO-R |
| *Bordetella* | RpoB_DPO1-F | RpoB_DPO-R |
| *Borrelia* | RpoB_DPO1-F | RpoB_DPO-R |
| *Brevibacillus* | RpoB_DPO1-F | RpoB_DPO-R |
| *Brucella* | RpoB_DPO1-F | RpoB_DPO-R |
| *Burkholderia* | RpoB_DPO1-F | RpoB_DPO-R |
| *Campylobacter* | RpoB_DPO1-F | RpoB_DPO-R |
| *Capnocytophaga* | RpoB_DPO1-F | RpoB_DPO-R |
| *Catonella* | RpoB_DPO1-F | RpoB_DPO-R |
| *Cereibacter* | RpoB_DPO1-F | RpoB_DPO-R |
| *Citrobacter* | RpoB_DPO1-F | RpoB_DPO-R |
| *Clostridioides* | RpoB_DPO1-F | RpoB_DPO-R |
| *Clostridium* | RpoB_DPO1-F | RpoB_DPO-R |
| *Colibacter* | RpoB_DPO1-F | RpoB_DPO-R |
| *Corynebacterium* | Mismatch | RpoB_DPO-R |
| *Cronobacter* | RpoB_DPO1-F | RpoB_DPO-R |
| *Cutibacterium* | RpoB_DPO1-F | RpoB_DPO-R |
| *Deinococcus* | RpoB_DPO1-F | Mismatch |
| *Desulfovibrio* | Mismatch | RpoB_DPO-R |
| *Dialister* | RpoB_DPO1-F | RpoB_DPO-R |
| *Dietzia* | RpoB_DPO1-F | RpoB_DPO-R |
| *Eggerthella* | RpoB_DPO1-F | RpoB_DPO-R |
| *Eikenella* | RpoB_DPO1-F | RpoB_DPO-R |
| *Enterobacter* | RpoB_DPO1-F | RpoB_DPO-R |
| *Enterococcus* | RpoB_DPO1-F | RpoB_DPO-R |
| *Escherichia* | RpoB_DPO1-F | RpoB_DPO-R |
| *Eubacterium* | RpoB_DPO1-F | RpoB_DPO-R |
| *Faecalibacterium* | RpoB_DPO1-F | RpoB_DPO-R |
| *Filifactor* | RpoB_DPO1-F | RpoB_DPO-R |
| *Finegoldia* | RpoB_DPO1-F | RpoB_DPO-R |
| *Fusobacterium* | RpoB_DPO1-F | RpoB_DPO-R |
| *Gemella* | RpoB_DPO1-F | RpoB_DPO-R |
| *Granulicatella* | RpoB_DPO1-F | RpoB_DPO-R |
| *Haemophilus* | RpoB_DPO1-F | RpoB_DPO-R |
| *Hafnia* | RpoB_DPO1-F | RpoB_DPO-R |
| *Helicobacter* | RpoB_DPO1-F | RpoB_DPO-R |
| *Jonquetella* | RpoB_DPO1-F | RpoB_DPO-R |
| *Kingella* | RpoB_DPO1-F | RpoB_DPO-R |
| *Klebsiella* | RpoB_DPO1-F | RpoB_DPO-R |
| *Lachnoanaerobaculum* | RpoB_DPO2-F | RpoB_DPO-R |
| *Lacticaseibacillus* | RpoB_DPO1-F | RpoB_DPO-R |
| *Lactiplantibacillus* | RpoB_DPO1-F | RpoB_DPO-R |
| *Lactobacillus* | RpoB_DPO1-F | Mismatch (some species) |
| *Lancefieldella* | RpoB_DPO1-F | RpoB_DPO-R |
| *Lawsonella* | RpoB_DPO1-F | RpoB_DPO-R |
| *Leptospira* | RpoB_DPO1-F | RpoB_DPO-R |
| *Leptotrichia* | RpoB_DPO1-F | RpoB_DPO-R |
| *Limosilactobacillus* | RpoB_DPO1-F | RpoB_DPO-R |
| *Listeria* | RpoB_DPO1-F | RpoB_DPO-R |
| *Mediterraneibacter* | RpoB_DPO1-F | RpoB_DPO-R |
| *Megasphaera* | RpoB_DPO1-F | RpoB_DPO-R |
| *Metamycoplasma* | Mismatch | RpoB_DPO-R |
| *Moraxella* | RpoB_DPO1-F | RpoB_DPO-R |
| *Morganella* | RpoB_DPO1-F | RpoB_DPO-R |
| *Mycobacterium* | RpoB_DPO1-F | RpoB_DPO-R |
| *Mycoplasmoides* | Mismatch | RpoB_DPO-R |
| *Neisseria* | RpoB_DPO2-F | RpoB_DPO-R |
| *Nocardia* | RpoB_DPO1-F | RpoB_DPO-R |
| *Odoribacter* | RpoB_DPO1-F | RpoB_DPO-R |
| *Olsenella* | RpoB_DPO1-F | RpoB_DPO-R |
| *Oribacterium* | RpoB_DPO1-F | RpoB_DPO-R |
| *Pantoea* | RpoB_DPO1-F | RpoB_DPO-R |
| *Parabacteroides* | RpoB_DPO1-F | RpoB_DPO-R |
| *Paracoccus* | RpoB_DPO1-F | RpoB_DPO-R |
| *Parvimonas* | RpoB_DPO1-F | RpoB_DPO-R |
| *Pasteurella* | RpoB_DPO1-F | RpoB_DPO-R |
| *Peptoniphilus* | RpoB_DPO1-F | RpoB_DPO-R |
| *Peptostreptococcus* | RpoB_DPO1-F | RpoB_DPO-R |
| *Phocaeicola* | RpoB_DPO1-F | RpoB_DPO-R |
| *Porphyromonas* | RpoB_DPO1-F | RpoB_DPO-R |
| *Prevotella* | RpoB_DPO2-F | RpoB_DPO-R |
| *Proteus* | RpoB_DPO1-F | RpoB_DPO-R |
| *Providencia* | RpoB_DPO1-F | RpoB_DPO-R |
| *Pseudomonas* | RpoB_DPO1-F | RpoB_DPO-R |
| *Raoultella* | RpoB_DPO1-F | RpoB_DPO-R |
| *Rhodobacter* | RpoB_DPO1-F | RpoB_DPO-R |
| *Rickettsia* | RpoB_DPO1-F | RpoB_DPO-R |
| *Roseburia* | RpoB_DPO1-F | RpoB_DPO-R |
| *Rothia* | RpoB_DPO1-F | RpoB_DPO-R |
| *Ruminococcus* | RpoB_DPO1-F | RpoB_DPO-R |
| *Saccharibacteria* (TM7) | RpoB_DPO1-F | Mismatch |
| *Salmonella* | RpoB_DPO1-F | RpoB_DPO-R |
| *Schaalia* | RpoB_DPO1-F | RpoB_DPO-R |
| *Selenomonas* | RpoB_DPO1-F | RpoB_DPO-R |
| *Serratia* | RpoB_DPO1-F | RpoB_DPO-R |
| *Shigella* | RpoB_DPO1-F | RpoB_DPO-R |
| *Slackia* | RpoB_DPO1-F | RpoB_DPO-R |
| *Sneathia* | RpoB_DPO1-F | RpoB_DPO-R |
| *Solobacterium* | RpoB_DPO1-F | RpoB_DPO-R |
| *Staphylococcus* | RpoB_DPO1-F | RpoB_DPO-R |
| *Stenotrophomonas* | RpoB_DPO1-F | RpoB_DPO-R |
| *Stomatobaculum* | RpoB_DPO1-F | RpoB_DPO-R |
| *Streptococcus* | RpoB_DPO1-F | RpoB_DPO-R |
| *Stutzerimonas* | RpoB_DPO1-F | RpoB_DPO-R |
| *Sutterella* | RpoB_DPO1-F | RpoB_DPO-R |
| *Tannerella* | RpoB_DPO1-F | RpoB_DPO-R |
| *Treponema* | RpoB_DPO1-F | RpoB_DPO-R |
| *Tropheryma* | RpoB_DPO1-F | RpoB_DPO-R |
| *Trueperella* | RpoB_DPO1-F | RpoB_DPO-R |
| *Tsukamurella* | RpoB_DPO1-F | RpoB_DPO-R |
| *Veillonella* | RpoB_DPO1-F | RpoB_DPO-R |
| *Yersinia* | RpoB_DPO1-F | RpoB_DPO-R |
