# Supplementary material for: Broad-range amplification and sequencing of the rpoB gene: a novel assay for bacterial identification in clinical microbiology
Source: J Clin Microbiol. 2024 Jun 17;62(7):e00266-24. doi: 10.1128/jcm.00266-24 (PMC11324016; doi:10.1128/jcm.00266-24)
Supplement: Table S2 — Positive and negative controls. [file jcm.00266-24-s0005.docx]

**SUPPLEMENTARY TABLE S2** Ct values of positive and negative extraction controls for *rpoB* and the 16S rRNA gene.

| **Run ID** | **Positive control** | **Negative control (bacterial colonies)** | **Negative control (clinical samples)** |
| --- | --- | --- | --- |
| 1 | 29,16 | 38,46 |  |
| 2 | 30,18 | 36,97 |  |
| 3 | 29,53 | 38,75 |  |
| 4 | 29,18 | 34,99 |  |
| 5 | 28,96 | 37,26 |  |
| 6 | 29,10 | 38,50 |  |
| 7 | 28,86 | 35,73 |  |
| 8 | 29,39 | no visible curve |  |
| 9 | 28,92 | 38,52 |  |
| 10 | 28,88 | no visible curve |  |
| 11 | 28,85 | 38,65 |  |
| 12 | 29,30 | 38,65 |  |
| 13 | 29,58 | 35,88 |  |
| 14 | 30,13 | 38,01 |  |
| 15 | 28,81 | no visible curve |  |
| 16 | 29,87 | 39,14 |  |
| 17 | 29,28 | no visible curve |  |
| 18 | 29,50 | 39,30 | 36,69 |
| 19 | 29,80 | 38,28 |  |
| 20 | 28,98 | 37,75 | 39,19 |
| 21 | 30,26 | 36,99 |  |
| 22 | 29,79 | 38,80 |  |
| 23 | 28,82 | 37,22 |  |
| 24 | 29,15 | 37,79 |  |
| 25 | 27,28 | 35,80 |  |
| 26 | 27,80 | 38,99 |  |
| **Average** | 28,48 | 38,72 | 37,94 |

***rpoB* PCR controls**

**16S rRNA PCR controls**^1^

|  | **Positive control** | **Negative control (bacterial colonies)** | **Negative control (clinical samples)** |
| --- | --- | --- | --- |
| **Average** | 22,36 | 33,09 | 32,71 |

^1^ This is an established routine method that is monitored according to our laboratory standards. Only average values are provided.
